# Patient-specific finite element modeling of scoliotic curve progression using region-specific stress-modulated vertebral growth

**DOI:** 10.1007/s43390-022-00636-z

**Published:** 2023-01-03

**Authors:** Christian R. D’Andrea, Amer F. Samdani, Sriram Balasubramanian

**Affiliations:** 1 School of Biomedical Engineering, Science and Health Systems, Drexel University, 3141 Chestnut Street, Bossone 718, Philadelphia, PA 19104 USA; 2 Shriners Hospitals for Children, Philadelphia, PA USA

**Keywords:** Adolescent idiopathic scoliosis, Patient-specific, Curve progression, Hueter-volkmann, Growth modulation, Skeletal maturity, Finite element model

## Abstract

**Purpose:**

This study describes the creation of patient-specific (PS) osteo-ligamentous finite element (FE) models of the spine, ribcage, and pelvis, simulation of up to three years of region-specific, stress-modulated growth, and validation of simulated curve progression with patient clinical angle measurements. *Research Question:* Does the inclusion of region-specific, stress-modulated vertebral growth, in addition to scaling based on age, weight, skeletal maturity, and spine flexibility allow for clinically accurate scoliotic curve progression prediction in patient-specific FE models of the spine, ribcage, and pelvis?

**Methods:**

Frontal, lateral, and lateral bending X-Rays of five AIS patients were obtained for approximately three-year timespans. PS-FE models were generated by morphing a normative template FE model with landmark points obtained from patient X-rays at the initial X-ray timepoint. Vertebral growth behavior and response to stress, as well as model material properties were made patient-specific based on several prognostic factors. Spine curvature angles from the PS–FE models were compared to the corresponding X-ray measurements.

**Results:**

Average FE model errors were 6.3 ± 4.6°, 12.2 ± 6.6°, 8.9 ± 7.7°, and 5.3 ± 3.4° for thoracic Cobb, lumbar Cobb, kyphosis, and lordosis angles, respectively. Average error in prediction of vertebral wedging at the apex and adjacent levels was 3.2 ± 2.2°. Vertebral column stress ranged from 0.11 MPa in tension to 0.79 MPa in compression.

**Conclusion:**

Integration of region-specific stress-modulated growth, as well as adjustment of growth and material properties based on patient-specific data yielded clinically useful prediction accuracy while maintaining physiological stress magnitudes. This framework can be further developed for PS surgical simulation.

## Introduction

Adolescent idiopathic scoliosis (AIS) is a complex three-dimensional (3D) deformity of the spine defined by a progressive frontal plane curvature and axial rotation affecting 1–3% of 10–16 year-olds in the US [1]. This condition has been associated with a perception of physical limitation and a decrease in self-esteem and body image [2]. The formation of lateral spine curvature observed in AIS is associated with alterations in the stress profiles across vertebral epiphyseal growth plates, which has been shown to alter local growth rates [3]. The Hueter-Volkmann law, the guiding principle of growth modulation in AIS spine, states that growth is stimulated in relative tension and inhibited in relative compression [4]. This law was validated across multiple species and anatomical locations, and has been shown to produce predictable alterations in bone growth when a known loading regimen is applied [5]. Quantifying the relationship between applied stress and resulting growth in the human pediatric scoliotic spine to predict curve progression on a patient-specific basis can assist in the optimization of the type and timing of intervention.

Finite Element (FE) methods using beam elements have been widely used to model asymmetric stress to simulate progressive AIS [6–12]. More recently, FE models consisting of both volumetric (tetrahedral or hexahedral) and beam elements have been reported, allowing for more detailed analysis of stress applied to the vertebral epiphyses [12, 13]. Compressive stresses in these volumetric models, based on gravity and muscle stabilization forces, determine growth modulation of vertebral body height [13]. While these models were validated with longitudinal clinical data from patients with progressive scoliosis, the precision of their predictions is limited by simplifications of vertebral growth that do not consider direction- and region-specific variations in growth rates for all anatomical regions of the vertebrae, including the posterior regions, through which 3–25% of longitudinal-axis compressive stress may be transferred [14, 15]. Recently published comprehensive data on region-specific normative pediatric vertebral morphology and size-invariant shape [16–18] have been used to validate region-specific orthotropic growth in a normative 10-year-old T1-L5 spine FE model [15]. Such methods and data could be used to incorporate asymmetric stress-modulated orthotropic region-specific growth in scoliotic spine FE models.

Maximizing the accuracy of material property assignment also plays a crucial role in simulation of stress-modulated growth; parameters such as intervertebral disc (IVD) elastic modulus and ligament stiffness may alter stress distribution at the vertebral epiphyses. While linear elastic material properties for vertebrae, intervertebral discs, and ligaments, have been implemented in several prior models, a recent study from our group used age- and level-specific non-linear mechanical properties for the spinal ligaments to improve model biofidelity [15, 19–21]. Additionally, since pediatric tissue properties are not widely available, age-based scaling methods have been applied to level-specific properties obtained from adult cadaveric specimen [22, 23]. However, no study has implemented age- and level-specific material properties in an osteo-ligamentous pediatric scoliotic spine FE model which incorporates region-specific, stress-modulated growth.

A longitudinal study from our lab illustrated significant differences in vertebral growth patterns between normative and scoliotic vertebrae in skeletally immature rabbits [24]. While comprehensive reporting of normative vertebral shape and morphology with growth has been reported, similar data are not available for the scoliotic vertebral growth in humans [16, 18, 25]. Additionally, variations (biological and/or inter-subject) in several prognostic factors including age, sex, weight, skeletal maturity, and spine flexibility, contribute to variable scoliotic curve progression [3, 26–28]. These prognostic factors have not yet been integrated together in an FE modeling framework of AIS with region-specific, stress-modulated vertebral growth; therefore, such an integration would significantly improve patient-specific modeling and prediction of curve progression [13, 29].

The objective of the current study is to simulate and validate region-specific stress-modulated vertebral growth in patient-specific scoliotic spine FE models which integrate age, sex, weight, skeletal maturity, and spine flexibility. Such FE models can serve as a tool to predict curve progression, and can also aid in decision making for clinical intervention. The current study will build upon our previously published work on pediatric patient-specific FE modeling and growth [15, 30].

## Methods

### AIS patient selection

After institutional review board approval, frontal and lateral low-dose bi-planar radiographs (EOS Imaging Inc, Paris) of 264 AIS patients were obtained from the Shriners Hospitals for Children, Philadelphia, PA, USA. The inclusion criteria for patient selection were: male and female AIS patients with skeletal maturity of Risser 0–4, with three years of follow-up (2.65 ± 0.30 years: 914, 1106, 1031, 994, and 785 days) at six-month intervals (average deviation of 31 ± 21 days), and either not braced or not brace-compliant. From this cohort, five AIS patients were selected, each having a unique Risser score of 0 through 4, respectively. These patients were either unbraced (three patients–1, 3, and 5), or non-compliant with brace wear (two patients–2 and 4), such that bracing effects were considered negligible. Table 1 shows a patient cohort that ranges in age, sex, Lenke type, Risser sign, initial and final Cobb angles, and spine flexibility, quantified via spine flexibility ratio (SFR), defined in Eq. 1.

**Table 1:** AIS patients for whom patient-specific FE models were created

Patient number	Initial Age (yrs), sex (M/F)	Lenke type	Initial Risser sign	Cobb angle (°) [Initial, Final]	Cobb angle during LB into Convexity (°)	Spine flexibility ratio (SFR)	Weight (kg)*
1	11 F	1AN	0	38.6, 46.3	13.7	0.70	37.0
2	11 F	1A-	1	42.5, 50.1	18.5	0.63	37.0
3	16 M	5CN	2	48.9, 52.9	23.2	0.56	60.7
4	13 M	1AN	3	39.5, 50.8	26.2	0.48	45.4
5	14 F	1CN	4	48.9, 52.9	36.8	0.30	49.2

*LB* lateral bending

*Patient weight was not provided in the current patient dataset, so the sex- and age-based 50th percentile weights from CDC growth chart were utilized [63]

### Vertebral geometry reconstruction from bi-planar radiographs and finite element mesh generation

A total of 153 vertebral landmark points (nine per vertebra) were selected in the frontal and sagittal radiographs and registered by triangulation using a custom code (MATLAB 2020b, MathWorks, Natick, MA) [15, 31]. 3D reconstructions of vertebrae using the landmark points were validated by comparing 3D landmark point locations obtained from frontal and lateral radiographs to those obtained directly from a chest CT scan. The average 3D reconstruction surface deviation was 3.0 ± 2.2 mm. Prior PS reconstruction methods reported similar accuracies [30, 32].

**Fig. 1:**
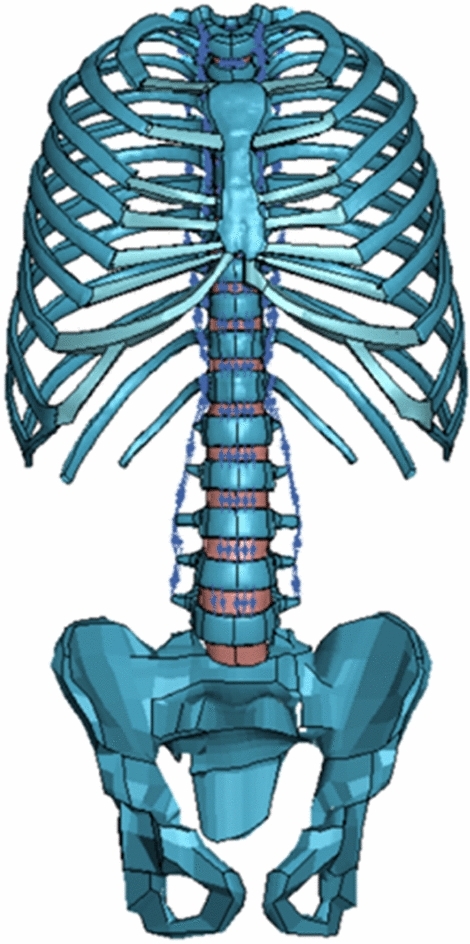
The template normative osteo-ligamentous FE model utilized to generate patient-specific models, which includes spine, IVD, ribcage, pelvis, and spinal ligaments

**Fig. 2:**
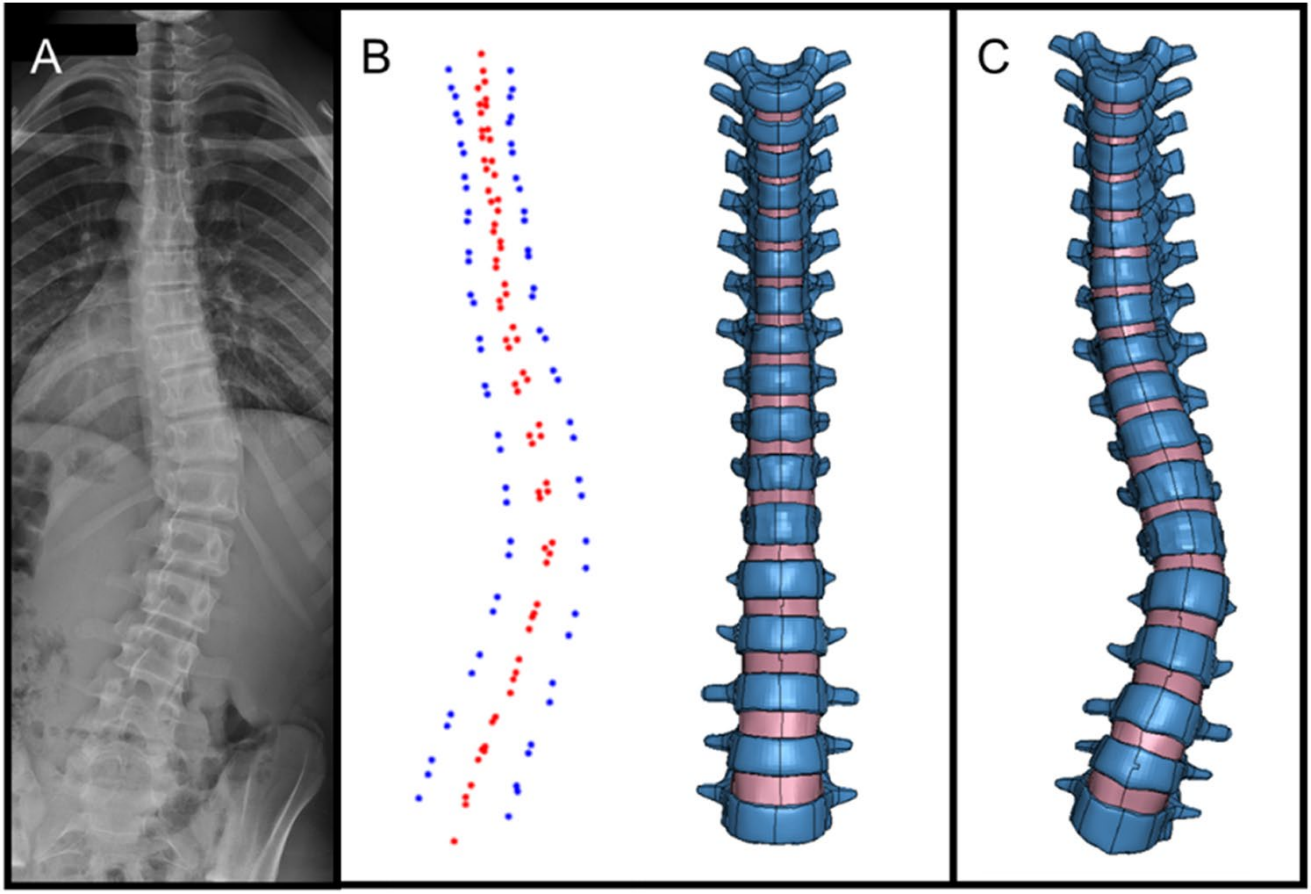
**A** Example (Patient 3) Frontal X-Ray, **B** Patient-specific landmark point selections (blue = frontal vertebral body corners, red = sagittal vertebral body corners), normative FE template model, **C**) Patient-specific FE model. In **B** and **C**, Ribcage, pelvis, and ligaments are hidden

For all subjects, after landmark point registration, PS-FE models were generated using a previously reported dual-kriging method, which morphs a normative FE spine model template (Fig. 1) to PS spine geometry based on vertebral landmark points (Fig. 2) [30, 33, 34]. The normative FE template utilized was a hexahedral FE model of a 10-year-old osteo-ligamentous thoracic and lumbar spine (T1-L5 with IVDs), ribcage, and pelvis with age- and level-specific ligament properties and orthotropic region-specific vertebral growth [15]. The costo-vertebral joints were modeled as beams constrained to tension–compression, and the pelvis was modeled as one unified structure, therefore no sacroiliac joint was separately modeled. This model consists of eight-node hexahedral elements with element side lengths ranging from 2 to 4 mm. After morphing the FE template model to each PS geometry, mesh quality was compared to previously established acceptability criteria metrics [30, 35].

### Assignment of patient age- and level-specific material properties

All anatomical structures aside from the ligaments (i.e., cortico-cancellous bone of the vertebrae, ribs, sternum, and pelvis, IVD, and costal, costo-vertebral, and transverse joint cartilage) were modeled as linear elastic materials, while all spinal ligaments were modeled as tension-only springs. Age- and level-specific material properties were assigned to the anatomical structures in each PS-FE model, shown in Table 2. The values for each mechanical property were obtained from published adult values which were adjusted based on chronological age-based scale factors [22, 36–40]. A comprehensive table of level-specific material properties of spinal ligaments are reported in the Appendix of previous work from our lab [15]. Furthermore, to increase patient-specificity of the FE model, IVD elastic modulus was scaled linearly with PS SFR (Eq. 1):

1 $$\mathrm{SFR}=\frac{\mathrm{CAS}-\mathrm{CALB}}{\mathrm{CAS}}$$

where SFR = Spine Flexibility Ratio, CAS = Cobb Angle while Standing (at Final Timepoint), and CALB = Cobb Angle during Lateral Bending into Convexity (at Final Timepoint).

**Table 2:** Age- and level-specific material property assignments

Anatomical structures	Adult elastic modulus (E, MPa) or stiffness (k, N/mm) [reference(s)]	Example Scale factor, patient 3 [22]	Example scaled elastic modulus (E, MPa) or stiffness (k, N/mm), Patient 3 [reference(s)]
Vertebrae	*E* = 350 [22, 36]	0.95	*E* = 332.5
IVDs	*E* = 20 [63]	0.95	*E* = 19.0
Spinal ligaments	Level-specific ligament properties [15]	0.97	Age-and Level-specific Force–deflection curves [15]
Ribs	*E* = 2100 [39]	0.95	*E* = 1995.0
Sternum	*E* = 2100 [39]	0.95	*E* = 1995.0
Costal cartilage	*E* = 10.4 [37, 38]	0.95	*E* = 9.9
Costo-vertebral & transverse joint cartilage	*k* = 48.9 [40]	0.94	*k* = 45.9
Pelvis	–	–	*E* = 5000 [63]

And the patient-specific, scaled elastic modulus of IVD, E_scaled_, was determined using Eq. 2:

2 $${E}_{\mathrm{scaled}}= {E}_{\mathrm{baseline}}\left(1-\mathrm{C}\times \mathrm{SFR}\right)$$

where E_baseline_ is the baseline elastic modulus of IVD (Table 2), and C is a constant, solved with iterative error minimization to be 0.3 [28].

### Asymmetric stress-modulated growth implementation

Vertebral growth was modeled through adaptation of a region-specific orthotropic thermal expansion method described by Balasubramanian et al. [15]. This method allocated 663 thermal expansion coefficients (for 17 vertebral levels × 13 regions × 3 directions), which were initially assigned values corresponding to normative age- and sex-based vertebral growth strains calculated with values obtained by Peters et al. [16, 17]. To represent the stress-modulated asymmetric growth that is observed in AIS patients, normative thermal expansion coefficients for vertical growth in vertebral bodies were scaled using compressive stress in the corresponding IVD region (i.e., anterior left vertebral body growth is scaled based on compressive stress in anterior left IVD region). Each growth rate was updated once per time interval documented for each patient. To achieve this scaling, a linear relationship between stress and growth rate was utilized, defined by Stokes et al. [3]. In Eq. 3, *G* is resultant growth (mm), G_m_ is baseline growth under normative stress (mm), $\beta$ is stress sensitivity (MPa^−1^), $\sigma$ is the altered stress (MPa), and ${\sigma }_{m}$ is the baseline stress (MPa). The value for $\beta$ was initially defined as 0.4 MPa^−1^ as reported by Shi et al. (2009), and decreased linearly according to Risser sign by the relationship $\beta =0.4-(\mathrm{Risser Sign}\times 0.08)$, such that as a patient approached Risser 5 (i.e., skeletal maturity), $\beta$ would approach zero [41].

3 $$G= {G}_{m}[1+\beta \left(\sigma -{\sigma }_{m}\right)].$$

### Boundary conditions (contacts and constraints), and loading conditions (gravitational force)

The pelvis was constrained in all translation and rotation directions, while the T1 vertebra was limited only to vertical translation. The articulating surfaces between facets were defined as sliding contacts with zero friction and exponentially increasing penalty force normal to any penetrating nodes until a maximum penetration depth (automatic LS-DYNA default, ex: half the element side length) was reached, while the interfaces between IVDs and vertebral endplates were defined as tied contacts. Gravity was applied through an adaptation of a method by Clin et al. (2011), wherein global vertical ‘anti-gravity’ forces were applied in the upward direction, all stresses were reset, and gravity forces of the same magnitudes were then applied in the downward direction, pairing spine positioning (standing) with a corresponding stress profile [26]. The magnitude of each force vector was determined based on percentage of bodyweight acting at each level, and applied from the geometric centroid of each vertebra. After gravity application, the total principal compressive stresses in each region were computed.

### Validation of spine curvatures and vertebral wedging with growth

Each patient-specific FE model was validated with measures of thoracic and lumbar Cobb angles, kyphosis, lordosis, axial rotation, and wedging of the apical vertebral body, at the time of reconstruction and at each subsequent yearly timepoint up to three years. A custom MATLAB code was created to extract these measurements at each reported timepoint, manually from each patient radiograph and automatedly from each patient-specific FE model. While mean inter-observer error in Cobb angle measurement on X-rays is generally reported as 3–5°, absolute error value threshold between radiograph-obtained measurements and those extracted from each PS-FE model was set at 8° for all angles, based on a reported 95% confidence interval for human error involved in manual angle extraction from radiographs [42, 43].

## Results

PS osteo-ligamentous FE models with ribcage and pelvis, with age- and level- specific material properties were generated for five AIS patients, stress-modulated growth was simulated at each radiograph acquisition timepoint, and FE simulation results were compared to data extracted from patient radiographs at each corresponding timepoint. For all FE models generated, each containing 307,564 hexahedral elements, mesh quality met the following previously reported acceptance criteria: Jacobian ≥ 0.5 (99.3% of all elements), aspect ratio ≤ 5 (99.7%), skewness ≤ 60° (99.2%), warpage ≤ 40° (99.6%), quadrilateral face minimum angle ≥ 30° (98.7%), and quadrilateral face maximum angle ≤ 150° (97.8%) [15, 30, 35, 44, 45].

### Spine curvatures

**Fig. 3:**
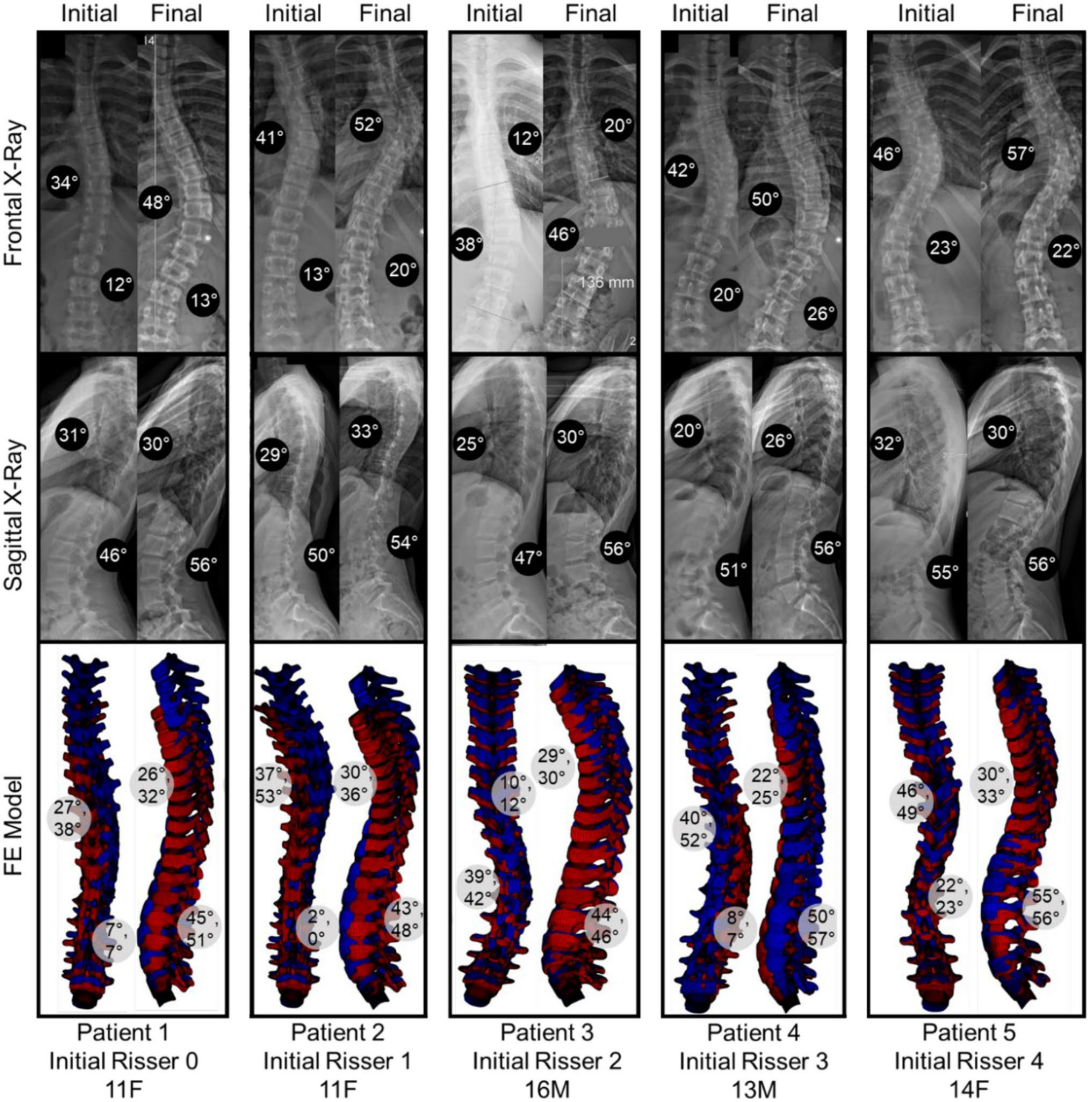
Comparison of clinical indices on patient frontal (first row) and sagittal (second row) X-rays and FE models (third row). Red models indicate initial timepoint, and blue indicate model solution after three years of simulated growth. In the third row, left models in each column are frontal views and right models in each column are sagittal views, and top degree values indicate measurements at zero years, while bottom degree values indicate measurements after three years of simulated growth. Ribs and ligaments have been removed for improved spine visibility

Average errors (defined as absolute values of error between FE model and X-Ray measured angle) in thoracic and lumbar Cobb angles were 6.3 ± 4.6° and 12.2 ± 6.6°, respectively, and average errors in kyphosis and lordosis angles were 8.9 ± 7.7° and 5.3 ± 3.4°, respectively (Fig. 3, Table 3).

**Table 3:** Average error between the current PS–FE models and patients’ clinical indices

	ITP	ITP + 1y	ITP + 2y	ITP + 3y	All TPs
Thoracic Cobb (°)	5.7 ± 4.4	5.5 ± 4.6	7.4 ± 5.5	6.2 ± 5.2	6.3 ± 4.6
Lumbar Cobb (°)	10.9 ± 5.2	11.4 ± 7.7	13.0 ± 6.9	13.5 ± 7.2	12.2 ± 6.6
Kyphosis (°)	8.8 ± 6.6	9.6 ± 7.6	7.7 ± 6.4	12.5 ± 11.3	8.9 ± 7.7
Lordosis (°)	8.2 ± 7.4	5.5 ± 3.0	7.0 ± 6.3	3.1 ± 1.8	5.3 ± 3.4
Axial rotation (°)	6.7 ± 6.6	7.6 ± 9.4	10.1 ± 10.9	12.3 ± 13.1	10.8 ± 11.1

*ITP*  initial time point

### Vertebral wedging

**Fig. 4:**
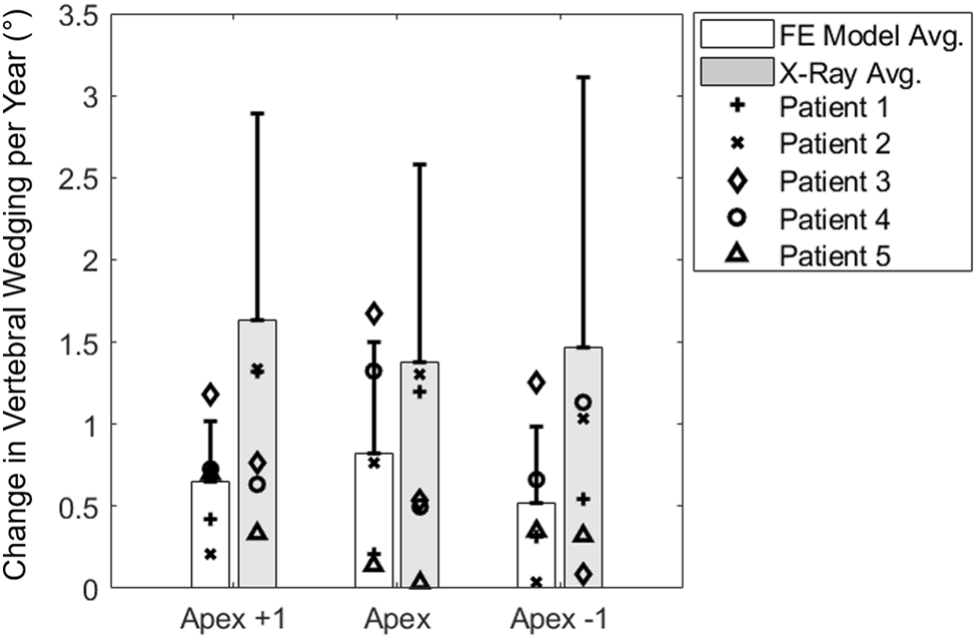
Comparison of change in wedging angle per year (in degrees) for the apical and adjacent levels, between FE models and patient X-rays. Mean and standard deviation (bars and error bars), as well as individual patient data (points) are supplied as described in the figure legend

Wedging angle between the superior and inferior faces of the apical and two adjacent vertebral bodies was predicted to within an average error of 3.2 ± 2.2° (Table 4). Furthermore, error in change in wedging angle per year was 0.99 ± 0.95°. The difference between PS-FE models and patient X-Rays is shown in Fig. 4.

**Table 4:** Average error in frontal vertebral wedging angle of the apical and adjacent above and below levels, between the current PS-FE models and patient X-Rays

	ITP	ITP + 1y	ITP + 2y	ITP + 3y	All TPs
Apex + 1 (°)	2.9 ± 2.1	2.9 ± 2.4	3.0 ± 2.3	3.9 ± 3.6	3.2 ± 2.7
Apex (°)	4.1 ± 1.7	2.9 ± 1.5	3.1 ± 1.5	3.4 ± 2.0	3.4 ± 1.8
Apex − 1 (°)	2.3 ± 2.1	3.0 ± 1.8	3.5 ± 1.7	2.4 ± 1.7	2.8 ± 1.9

### Stress in IVDs

Average principal stress in each IVD region for the apical vertebral level (IVD below apical vertebra) is shown in Table 5 for each patient-specific FE model.

**Table 5:** IVD quadrant principal stresses at ITP and each subsequent yearly timepoint up to three years, as well as the overall change in component principal stress that occurred during simulated growth. Positive values indicate compression and negative values indicate tension

	ITP	ITP + 1y	ITP + 2y	ITP + 3y	$\Delta$
Anterior, convex (MPa)	0.34 ± 0.10	0.06 ± 0.14	0.04 ± 0.16	0.00 ± 0.17	− 0.34 ± 0.07
Anterior, concave (MPa)	0.34 ± 0.10	0.66 ± 0.10	0.74 ± 0.09	0.79 ± 0.11	0.46 ± 0.01
Posterior convex, (MPa)	0.20 ± 0.10	− 0.07 ± 0.05	− 0.09 ± 0.06	– 0.11 ± 0.07	− 0.31 ± 0.03
Posterior, concave (MPa)	0.20 ± 0.10	0.49 ± 0.24	0.73 ± 0.30	0.73 ± 0.32	0.54 ± 0.21

## Discussion

This is the first study to implement orthotropic region-specific stress-modulated growth in patient-specific FE models of the osteo-ligamentous T1-L5 spine, ribcage, and pelvis, with vertebra, ribcage and pelvis comprised entirely of volumetric elements, and with age- and level- specific material properties.

The absolute error values in clinical indices measured during and after three years of simulated growth were under 8°, 10°, and 15° in 60%, 72%, and 83% of angular measurements, respectively, across all patients and timepoints. For thoracic or thoraco-lumbar Cobb angle in particular, these percentages were 63%, 84%, and 90%, respectively. Yearly main thoracic or thoraco-lumbar curve progression rate was assumed to vary in each FE model with age, sex, Lenke type, Risser sign, initial and final Cobb angles, and SFR, and yearly FE model curve progression averaged 3.2 ± 5.4°/year. The observed FE model Cobb angle progression rates were considered realistic based on yearly curve progression rates observed in both the patient radiographs accessed for the current study, and on report of median AIS curve progression rate being approximately 7°/year [46]. Previous reports of apical vertebral wedging in silico from 0.6 to 1.4°/year corroborate the results of the current models. The corresponding average values in the current study ranged from 0.5 to 0.8°/year at the apical and immediately adjacent levels [29]. Furthermore, average vertebral column stress ranged from 0.11 MPa in tension to 0.79 MPa in compression, similar to the stress range of 0.3 MPa in tension to 0.7 MPa in compression reported by Clin et al. (2011) [26], though it should be noted that the prior study reported stresses for the entire T1-L5 spine, while the stress magnitudes reported in the current study consider only the apical and adjacent levels. Together, these results suggest that the current modeling approach can predict alterations in spine geometry and related vertebral wedging within the patient cohort with clinically useful accuracy.

In previous work from our lab, a sensitivity analysis on vertebral growth that varied ligament stiffness, IVD elastic modulus, bone elastic modulus, and thermal expansion coefficient was performed, indicating that IVD elastic modulus had the greatest effect on average stress magnitude measured in the IVD, and thermal expansion coefficient had the greatest effect on vertical vertebral body strain [33, 34]. Since thermal expansion coefficients cannot be varied directly, stress sensitivity was chosen as its proxy. Hence, stress sensitivity and IVD elastic modulus were selected for scaling in the current modeling framework. To assess the effects of scaling these two parameters on model outcomes, simulations of spine growth for one exemplar patient (patient 3) were also performed with these two parameters held constant independently. When each of these two parameters were held constant (i.e., not scaled), progression of thoracic Cobb, lumbar Cobb, kyphosis, lordosis, and axial rotation angles were affected. When stress sensitivity was not scaled, 0.59 ± 0.32, 0.54 ± 0.47, 1.09 ± 0.89, 0.24 ± 0.20, and 0.15 ± 0.13 degree differences occurred, respectively, and when IVD elastic modulus was not scaled 0.45 ± 0.23, 0.40 ± 0.31, 0.73 ± 0.71, 0.27 ± 0.20, and 0.24 ± 0.12 degree differences occurred, respectively. These sensitivity analyses justify the utility of scaling stress sensitivity based on patient skeletal maturity, and IVD elastic modulus based on patient flexibility.

The current FE model is limited by being solely osteo-ligamentous—lungs, muscles, and connective tissue beyond the intervertebral spinal ligaments were not included. These features, if included in future analyses, may alter the stress environment of the spine as a whole, and therefore alter the calculated stress sensitivity that would accurately represent curve progression. However, the stress sensitivity parameters calculated in the current FE model may be considered to compensate for its limited scope. Second, due to the relative coarseness of the Risser sign (0–5, in steps of 1) to chronological age (continuous), scaling of stress sensitivity based on a correlation between Risser sign and chronological age may contribute to error in prediction of curve progression. This limitation could be addressed by correlating a more precise (Sanders score) or even continuous scale (Collagen X biomarker levels) of skeletal maturity with chronological age, and using this relationship to more accurately scale stress sensitivity for each patient [47–49]. Third, IVD elastic modulus was scaled from normative data for all simulated timepoints based on spine flexibility obtained at the final timepoint (approximately 3 years after ITP), where in reality, IVD elastic modulus may vary between normative and AIS patients, and spine flexibility may not remain constant over this timespan [50]. Future studies may define IVD material property differences between normative and AIS patients. Additionally, IVD elastic modulus may be updated at each timepoint according to spine flexibility ratio by obtaining lateral bending imaging at those respective timepoints. Fourth, the growth of the ribcage and pelvis were not simulated in the current study. The purpose of the ribcage and pelvis in the current model is to accurately represent more biofidelic and anatomically correct loading and boundary conditions. Inclusion of the ribcage and pelvis would also be essential to assess range of motion of the model. The model could be improved in the future by implementing growth of other anatomical structures. Fifth, the effect of activity levels resulting from various exercise regimens on both reducing curve progression or decreasing an existing Cobb angle is considered significant based on a review of 19 publications [51]. However, the activity levels of the patients included in this study are unknown.

Sixth, stress effects on growth introduced by bracing were not accounted for, though brace wear compliance was reported to be low in the clinical notes and therefore considered negligible [52]. Further improvements to the modeling framework can consider vertebral stresses induced by externally applied loads from bracing. No patient activity level data were obtained, which may act as a source of prediction error. Inclusion of such data may prove to be a significant feature in precise and accurate curve progression. Seventh, while sex-specific normative baseline growth rates have been established, these baseline growth rates were assumed to apply to AIS patients. Eighth, genetic factors were not integrated in the current FE modeling approach. Numerous factors have been shown to correlate with AIS development and progression, and therefore future modeling framework development may benefit by integrating such relationships [53, 54]. Ninth, no sex-specific stress sensitivity or rate-of-change of stress sensitivity has been established [55]. While the effect of hormone levels has been shown to alter growth plate activity, longitudinal data on hormone levels in AIS patients is not typically collected, but may improve prediction accuracy [56]. Additionally, higher prediction errors of compensatory curve progression as compared to that of the primary curvature may be attributed to currently used optimization methods for stress sensitivity scaling, which only minimized error in primary curve progression. Error in compensatory curve progression prediction could be reduced in future studies by implementing alternative stress sensitivity scaling optimization methods. Lastly, no reduction in kyphosis was observed during curve progression in the current FE models, where such reduction was observed in a patient cohort [57]. In future analysis, this unmatched trend may be addressed through either compensatory force application to represent any currently missing model scope, or through altering sagittal growth modulation characteristics.

The modeling techniques developed in the current study may provide improved insights into the prediction of scoliotic curvature progression in AIS patients. This curve progression prediction utilizing increased detail of both local growth and internal stress can be developed further to assist in surgical timing, planning, and prognosis for growth-modulating interventions such as anterior vertebral body tethering (AVBT). Future models may benefit from accounting for effects of active muscle loading, overall thorax stiffness, including other structures such as lungs, diaphragm etc., and a more precise classification of deformity [58–61]. Additionally, anatomically complete FE models of progressive AIS can serve as a foundation for testing growth-modulation devices, as current human cadaveric spines or surrogates cannot mimic the growth patterns and biomechanical responses observed in children and adolescents, as well as to do so with large animal models used for device testing [24, 62, 63]. In a broader sense, predictive biomechanical models such as that developed in the current study can contribute to advancements in precision medicine and optimized clinical outcomes [64].

## Data Availability

The data used to support the findings of this study are available from the corresponding author upon request.
